# Wearable and Implantable Sensors: The Patient’s Perspective

**DOI:** 10.3390/s121216695

**Published:** 2012-12-05

**Authors:** Jeroen H. M. Bergmann, Vikesh Chandaria, Alison McGregor

**Affiliations:** 1 Imperial College London, Medical Engineering Solutions in Osteoarthritis Centre of Excellence, London W6 8RF, UK; 2 Imperial College London, Department of Bioengineering, London SW7 2AZ, UK; E-Mail: vikesh.chandaria08@imperial.ac.uk; 3 Imperial College London, Department of Cancer & Surgery, London W6 8RF, UK; E-Mail: a.mcgregor@imperial.ac.uk

**Keywords:** medical sensors, wearable technology, body sensor networks, patient centered design

## Abstract

There has been a rising interest in wearable and implantable biomedical sensors over the last decade. However, many technologies have not been integrated into clinical care, due to a limited understanding of user-centered design issues. Little information is available about these issues and there is a need to adopt more rigorous evidence standards for design features to allow important medical sensors to progress quicker into clinical care. Current trends in patient preferences need to be incorporated at an early stage into the design process of prospective clinical sensors. The first comprehensive patient data set, discussing mobile biomedical sensor technology, is presented in this paper. The study population mainly consisted of individuals suffering from arthritis. It was found that sensor systems needed to be small, discreet, unobtrusive and preferably incorporated into everyday objects. The upper extremity was seen as the favored position on the body for placement, while invasive placement yielded high levels of acceptance. Under these conditions most users were willing to wear the body-worn sensor for more than 20 h a day. This study is a first step to generate research based user-orientated design criteria’s for biomedical sensors.

## 1. Introduction

Technology has become an integrated part of medicine and it often defines how patients are diagnosed and treated. Medical devices have been at the forefront of many success stories, but at the same time advances in medical technology have contributed to the overall rising health care spending. The main drivers for this growth in spending have been the increased utilization of health care goods and services, as well as their associated cost [1]. National health spending in the USA alone reached $2.6 trillion in 2010. This money is divided over many stakeholders and the affiliated medical technology market is currently estimated to be worth £150–170 billion worldwide. It is predicted that the market size will approach £300 billion by 2015, with growth rates forecast at 10% per annum over the next five to six years [2]. A large part of that money will be directly spent on research and development within the medical device industry. One of the top priorities for many companies is the development of mobile healthcare devices. Increased mobility of healthcare delivery through wearable medical sensors has also gained the interest of many clinicians and researchers [3]. It has become a fast emerging research field, which promises to improve the quality of life for many patients, whilst reducing the cost of care [4]. Wearable and even implantable devices that monitor, administer, treat and track patient conditions are increasingly replacing larger instruments [5].

The progress made in nanoelectromechanical systems (NEMS) for mass spectrometry [6], patches for controlled drug delivery [7] and carbon nanotube sensors that can detect human movement [8] illustrates the research focus on ambulatory screening and therapy. Physiological signals can now be easily monitored using wearable or implantable sensors. This kind of biotechnology may have important clinical benefits, but at the same time it is also becoming an ever more demanding part of our lives and one that requires direct user engagement [9]. Thus, user preferences need to be taken into account if the next generation of systems are to gain acceptance in both a clinical and home setting [10]. The make or break question is often; how will patients respond to it? It is this question that is often not asked, as new technologies emerge at a rapid pace. Not surprisingly, there is a strong contrast between the amount of research conducted in this area and the amount of wearable sensor technology that ends up in clinical practice.

The aim of this study is to identify trends in user preferences for medical wearable sensing devices in a specific patient population. The term wearable sensing device for medical applications refers to any system that is connected to the body and measures clinical relevant information. The main focus of this study was on people suffering from osteoarthritis, as it is currently one of the key global medical issues associated with our ageing population. It is also a population that will use both non-invasive and invasive (implantable) wearable systems as part of their treatment regime. This particular sample population is likely to skew the results towards older females [11].

## 2. Methods

A recent systematic review could not find any published studies that used detailed research methods to investigate user preferences of wearable sensors [10]. Thus, a questionnaire was developed and validated to obtain relevant information regarding these preferences.

### 2.1. Subjects

This study targeted people aged 18 years or older. No further exclusion criteria were selected to guarantee the highest possible number of responders. Only those who completed 50% or more of the questionnaire were selected for further data analysis. The questionnaire was placed on www.oastudy.org and subjects were actively sought by using the University database, large arthritis charities and discussion forums for patients. The method of recruiting shows a focus on the UK population, with a strong (osteo) arthritis presence.

### 2.2. Questionnaire

The questionnaire was designed as a structured self-complete online questionnaire. The questionnaire was broken down into the six specific sections; an introduction, what the system should measure, how the device should look, previous knowledge of these devices, specific questions on device features and demographics (see Appendix of this document for the complete questionnaire). This order of questions was selected, as it is known that a better completion rate is achieved if general questions precede specific questions, with more personal questions placed at the end of the questionnaire [12]. The first part of the questionnaire consisted of open-ended questions, allowing for meaningful answers based on the respondents own knowledge and emotions. Participants were asked not to limit themselves to the abilities of current technology and assume that anything is possible. This ensured that the features described could inform us about new prospective design aspects, which we could incorporate into our development process.

The total completion rate is likely to be influenced by the open-ended questions at the start of the questionnaire. It is known that more responders abandon the questionnaire during open-ended questions compared to closed [13]. However, the open-ended questions provide a richer insight into the user preferences and generate more meaningful answers.

The section that contained specific closed-ended questions included previously identified preferences found in the literature [10]. These preferences need to be further assessed, as they were identified by a population often consisting of only a few subjects. The design feature section quantified the importance, according to the participant, of preferences and requirements previously identified in the literature.

**Figure 1.**
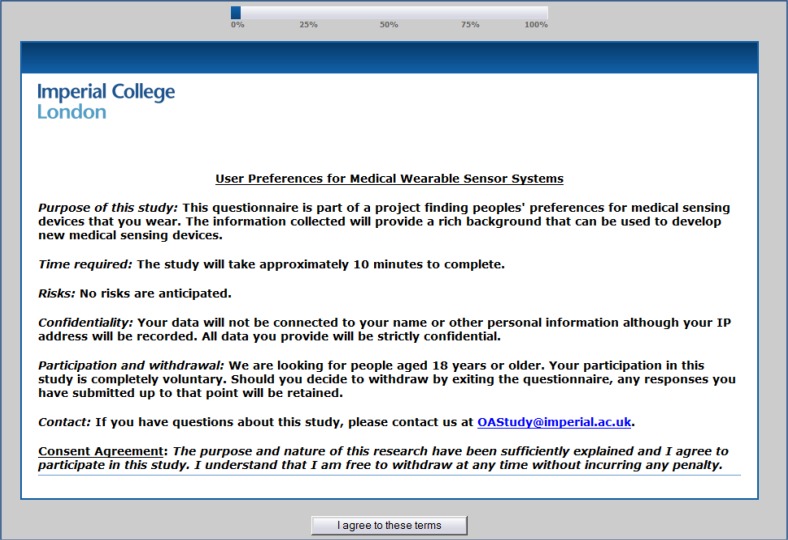
Screenshot of the opening page of the online questionnaire.

The first part of the questionnaire included an information sheet and a consent agreement. The information sheet informed the participant of the purpose of the study and the estimated time it will take to complete the questionnaire. Further explanation was also given regarding confidentiality, data collection, privacy and how to contact the researchers. All subjects had to give informed consent before they could participate in this research project. The lay-out and navigation of the online questionnaire was checked by an independent marketing research expert (Figure 1).

### 2.3. Validation of the Questionnaire

The questionnaire incorporated previous identified user issues for wearable devices [10], as well as potential relevant topics that were identified by the authors. Two colleagues were invited to participate on a pre-pilot study to determine if the questions were unambiguous, appropriate and acceptable to potential respondents. Subsequently, a small qualitative study was undertaken to further develop and validate the questionnaire. Short interviews were conducted to get views of experts on potential patient preferences that should be included in the survey. A medical doctor with expertise in patient reported outcomes, a musculoskeletal physiotherapist and three occupational therapists were interviewed regarding the user preferences of wearable sensor devices. All interviews were transcribed and the face validity of a questionnaire was examined by asking them afterwards to complete the questionnaire. The outcomes from the questionnaires matched the identified factors of the transcribed interviews. In addition, the questionnaire was tested in a focus group with six patient volunteers. All the relevant opinions and questions gathered in the focus group were used to further adjust the questionnaire. Questions that were unclear to the patients were adapted until all patients understood the question. The criterion validity could not be assessed, as there is currently no other existing scale that could act as comparison.

### 2.4. Reliability of the Questionnaire

The test–retest reliability was assessed by asking five people to complete the questionnaire again. At least three weeks separated both time points. Participants were asked to state any relevant change in circumstances, since the initial completion of the online questionnaire. Subjects that mentioned any meaningful changes were excluded from the reliability study in order to limit any potential bias in the test-retest outcome. However, none of the five subjects reported any significant changes in their situation. The open ended questions were qualitatively compared and it was found that 20% percent of all answers differed from those initially given by the responders. The majority of changes related to their opinion on how to prevent, diagnose and treat the medical condition. Questions that allowed for “yes” or “no” (and “external” or “internal”) answers showed an agreement of 95%. This indicates the response given for these polar items was very consistent. A weighted Kappa (using linear weighting) was used for the questions that generated categorical data. Most of this data was generated on a 3-point Likert scale with an additional “don’t know” option or on a 10-point Likert scale. The 3-point Likert scale showed a “moderate” Kappa of 0.55 for all questions combined, while a Kappa of 0.482 was found for the 10-point scale.

### 2.5. Acceptability of the Questionnaire

The Gunning Fog Index was used to measure the readability of the questionnaire. This index is based on the length of sentences and the difficultly of the words used. A score of 12 and higher is normally not suitable for a wide audience [14]. The questionnaire achieved an overall score of 9.7.

The time to completion was approximately 10 min. We aimed for it to be within the region of 10 min, as it is known that a short web survey that takes 8–10 min to complete has a lower non-response rate than those that take longer [13,15]. The questionnaire was specifically developed to stay within this predetermined completion time. Information regarding the estimated duration was included in the introduction page of the online questionnaire. A progress indicator was placed at the top of the page to inform the respondent how much of the questionnaire had been completed.

### 2.6. Database Review and Analysis

The database was reviewed to identify inconsistencies in the answers given for the open-ended questions. The open-ended questions were corrected for spelling mistakes, plural and singular variations to allow for a correct computation of frequencies. All words were given in lower-case to ensure bundling was accurate. Common words (e.g., conjunctions or adverbial genitive) were also removed to prevent these from showing up in the frequency tables and displays. A descriptive frequency method was applied to all the answers. The responses to open-ended questions were analysed using a cloud tag method to allow for a breakdown according to frequency [12]. The results were displayed as word clouds to provide a simple visual representation of word frequency.

### 2.7. Ethical Approval

The protocol was approved by the College Research Ethics Committee (ICREC_11_5_5) and all subjects gave informed consent previous to filling in the online questionnaire.

## 3. Results

In total 819 people visited the website, but only 299 (37% of the total) of them completed more than 50% of the questionnaire. Data of responders who completed more than 50% of the questionnaire were used for further processing. This yielded a completion rate of 37%.

### 3.1. Demographics

**Figure 2.**
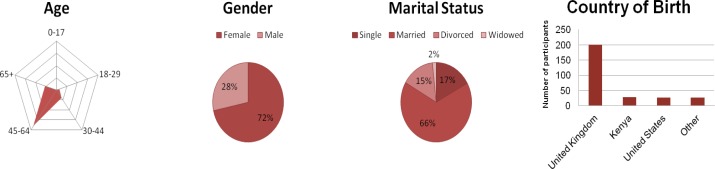
Demographics of the participants. Non-responders were not taken into account in these plots.

The mean age of the sample population was 54 years (range 18–79; standard deviation 12.5). The age group between 45 to 64 years showed the highest percentage of responders (Figure 2). In total 207 responders were female, 82 were male and 10 responders did not provide any gender information. Most responders were married and born in the United Kingdom.

**Figure 3.**
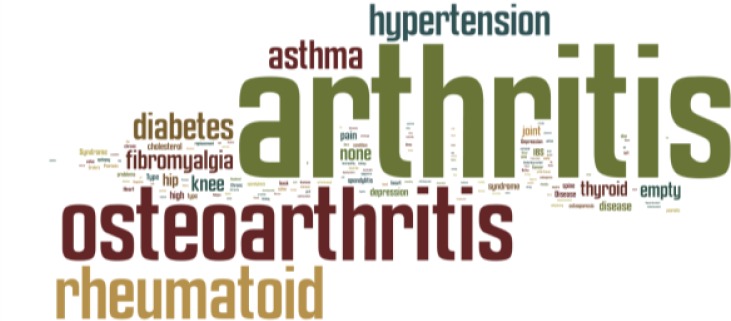
Medical conditions participants suffered from. The word cloud provides a graphical representation of frequency of words.

When participants were asked if they suffered from any medical condition (Figure 3), the majority mentioned some type of arthritis (52%). The second most common answer given was hypertension (12%), followed by asthma (11%) and diabetes (10%).

**Figure 4.**
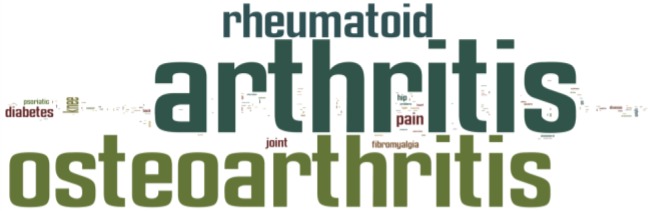
Medical condition people wanted to focus on. The word cloud provides a graphical representation of frequency of words.

All subjects were asked to state the medical condition they wanted to focus on. In this particular study, participants choose to answer the questionnaire with a main focus on arthritis (Figure 4).

Of the total number of responders, 27% of participants had prior knowledge of wearable medical sensing devices. However, only 5% of the subjects had any direct user experience with wearable medical devices. These experiences related mainly to heart problems (e.g., pacemaker) and diabetes (e.g., insulin pump).

### 3.2. Response to Open-Ended Questions

**Figure 5.**
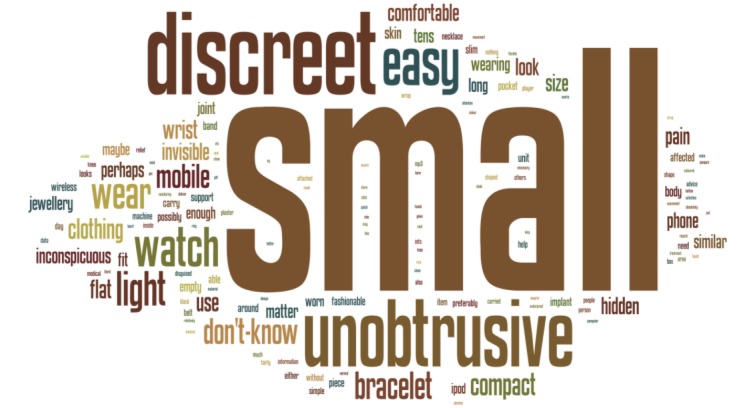
Word cloud generated for the question of how a wearable device should look. The cloud provides a graphical representation of the frequency at which specific words are used.

Data showed that the responders would prefer a small, discreet and unobtrusive system with many people referring back to everyday objects (as shown in Figure 5).

A closed-ended-question that incorporated specific follow-up questions was introduced in the open-ended section of the questionnaire and it focused on the acceptance of invasive sensing. The majority of people (∼85%) preferred the sensors to be worn external (non-invasive) to the body. However, when the group who preferred a non-invasive system was asked if they would wear an internal device in a potential life saving situation most people would. Only 14 people (∼5% of total population) stated that they would not want an implantable device in that specific situation. This topic was repeated in the closed-ended section, without fellow-up items and rephrased as implantable sensor.

When the participants were asked where they would like to wear the device 255 (85%) answered external, 21 (10.5%) said internal and 13 people (4.5%) left it blank. Table 1 provides the full breakdown of answers when participants were asked about how the device should be controlled.

Table 2 provides further detailed information on the responses regarding where subjects would like the device to be worn. Sections of the upper limb were mentioned in 38% of the cases. The torso and neck was given 30.8% of the time, while the lower limb reached a 22.5% response rate.

A median annual spend of £50 was found for the biotechnology that related to their own preference. The conversion rates given by the International Monetary Fund (IMF) for the 30th of March 2012 were used to convert all currencies to UK pound sterling.

### 3.3. Response to Closed-Ended Questions

The results for the closed-ended questions are given in Table 3. It showed that people are willing to spend time learning how to use a new wearable device. It also demonstrates that just over one third of the responders would wear an implantable device. This question does not contain any further specification of use (e.g., in a life saving situation) and the answer given therefore depends on the subjects own interpretation of the circumstances during which this might be needed.

A difference between genders of more than 5% was found for question 1, 3, 8, 13 and 14. Men were more likely to say yes in question 1, 3, 6 and 11, while a higher percentage of women answered “Yes” in question 10. However, for all these questions men also had the highest percentage of responders answering “No”.

A total of 62% of the people were willing to wear the device for more than 20 h a day. However, 37% did expect it to have a battery life of more than 6 months. The placement of the technology on or in the body is expected to take less than 5 min (59% of the overall number of replies) and 35% of the responders even thought it should be less than 1 min.

Participants were subsequently asked to rate several specific features on a 10 point scale (Table 4). The items tested contained some preferences that were previously highlighted by the literature [4] and some that were identified in a pilot focus group that consist of medical professionals. The highest rated item was that the device should be reliable and comfortable. People seemed to be the least concerned with the device being recyclable, although it still yielded an average importance value of 7.4. The observed variation does indicate a strong overlap between the ratings of all the statements.

## 4. Discussion

A range of themes regarding user preferences were covered to identify trends in patient preferences for medical wearable sensing devices. Some of the questions showed a clear preference, while responders were less homogeneous in their responses on other topics. Control of the device seemed a difficult question, as a quarter of all participants did not know how they wanted to control the technology, although control by using just a button was seen as a popular option (∼17%). When asked about placement on the body, the wrist (∼19%) or arm (∼14%) appeared to be the preferred location. More than half of all subjects were willing to wear the device for more than 20 h a day, but they did want the placement of the device to take less than 5 min, while the technology itself should have a running life of more than six months. The device itself should be small, unobtrusive and preferentially incorporated into everyday objects.

To our knowledge this is the first academic study that aims to gather the opinions of potential users on new biomedical sensors that you can “wear”. As can be expected, generalizability of these results is limited due to the demographics of the sample population. Further research is needed to explore other patient groups and confirm the present findings. However, this is a first step to generate more academic user orientated design criteria’s for this kind of biotechnology. The obtained information can be used to improve the design and development of novel body-worn sensor systems. This data also provides a preliminary insight into the likely acceptance of new nanosensors that measure pathologically elevated levels of disease biomarkers in for example osteoarthritis.

## 5. Conclusions

This study promotes that developers should consider their target user group at an early stage in the design process. It also brings attention to the need to rely more on objectively obtained data sets throughout the development process. Poor reporting on user preferences in this quickly growing research field has so far limited the build up of crucial knowledge needed for a more successful integration of these sensor technologies at a clinical stage. A reduction of the overall research and development cost, as well as increasing ecological utility can be achieved by implementing key design features for end-users at an early stage.

## Figures and Tables

**Table 1.** Percentage of responders providing a specific response to the question how they would like to control the wearable device. The table only includes answers that had a response rate of 1% or above.

**Means of control**	**Percentage**
Don’t know/unclear	24.7
By button	17.7
By remote control	15.1
Manually	10.4
By touch control	6.7
Automatic	5.4
By connection to a PC	3.3
Through smart phone or hand held device	3.0
By wireless control	2.3
By sensors	2.3
By voice	2.0
By switch	1.7
No control	1.3
By brain waves	1.0

**Table 2.** Percentage of responders providing a specific response to the question of where they would like to wear the technology. The table only includes answers that had a response rate of 1% or above.

**Means of control**	**Percentage**
Wrist	18.7
Arm	14.0
Anywhere	11.0
Torso/Abdomen	10.7
Waist	10.4
Leg	5.4
Hip	5.4
Neck	5.0
On the location of the medical condition	5.0
Knee	4.7
Back	4.7
Hand	4.0
Ankle	2.7
Thigh	2.3
Foot	2.0
Upper Body	1.7
Shoulder	1.3

**Table 3.** Percentage of participants that provided a specific categorical response to a closed question. Answers are given on a 3-point Likert scale, with an additional “don’t know” option.

**Question**	**Yes**	**Maybe**	**No**	**Don’t know**	**Left blank**
1. Would you wear a device that is visible to others?	32.4	11.7	51.8	3.0	1.0
2. Would you wear a device that is concealed in your clothing?	89.0	1.0	8.0	0.7	1.3
3. Would you wear an implanted device?	37.5	14.0	35.8	11.0	1.7
4. Would you allow continuous monitoring 24 h a day?	71.6	2.3	23.1	1.7	1.3
5. Would you allow data to be sent away and analysed or stored on a database?	80.6	2.3	14.0	1.7	1.3
6. Would you consider wearing a device to monitor how active you are?	78.3	2.7	15.4	2.0	1.7
7. Would you use a device that you had to apply yourself without assistance?	80.6	1.3	15.4	1.3	1.3
8. Would you like the device to determine the level of your condition (e.g., mild or severe)?	84.6	1.0	10.0	2.7	1.7
9. Would you wear a device that would tell the clinician if you did not wear it often enough?	76.9	3.3	17.1	1.7	1.0
10. Would you spend time learning how to use a new wearable device?	91.0	1.0	6.0	0.7	1.3
11. Would you wear an implant that has a sensor incorporated in it?	52.2	10.7	28.8	7.0	1.3

**Table 4.** Percentage of responders providing a response on a 10-point Likert scale to the questions that relate to the measuring system. Participants were asked on a scale of 1 to 10 how important the following statements were, with 10 being the highest.

**A medical sensing device that you wear should...**	**mean**	**Standard deviation**
...be comfortable	9.6	1.2
...be compact (light and small)	9.1	1.5
...be discrete	8.5	1.9
...be easy to attach to the body	9.1	1.4
...be recyclable	7.4	2.8
...be reliable	9.6	1.2
...be simple to operate (and maintain)	9.3	1.4
...blend in with different types of clothing to be worn with the device	7.6	2.4
...give instant feedback	8.1	2.2
...have clear and readable instructions	9.3	1.3
...increase the accuracy of current clinical procedures	9.3	1.3
...minimize incorrect use of the system	9.1	1.5
...motivate the people using it	8.8	1.6
...not affect normal daily behaviour	9.1	1.6
...not detach from patient unless needed	8.5	2.0
...provide clear and useful results	9.5	1.2
...provide positive feedback to patients	9.3	1.3
...reduce travel to clinics and hospitals	9.1	1.6
...speed up currently used clinical procedures	9.1	1.4
...work alongside your medical care team, instead of replacing them	9.2	1.4

## Appendix—Questionnaire

### Introduction

**Table:**

*Purpose of this study:* This questionnaire is part of a project finding peoples’ preferences for medical sensing devices that you wear. The Information collected will provide a rich background that can be used to develop new medical sensing devices.
*Time required*: The study will take approximately 10 minutes to complete.
*Risks*: No risks are anticipated.
*Confidentiality*: Your data will not be connected to your name or other personal information although your IP address will be recorded. All data you provide will be strictly confidential.
*Participation and withdrawal*: We are looking for people aged 18 years or older. Your participation in this study is completely voluntary. Should you decide to withdraw by exiting the questionnaire, any responses you have submitted up to that point will be retained.
*Contact* If you have questions about this study, please contact us at OAStudy@imperial.ac.uk.
**Consent Agreement:***The purpose and nature of this research have been sufficiently explained and I agree to participate in this study. I understand that I am free to withdraw at any time without incurring any penalty*.
**I agree to these terms**

**Table:**

The term medical sensing device refers to any system that is worn by the patient and measures clinically relevant information. These devices can be used to diagnose, prevent and treat disease. The devices provide healthcare that is specific to each patient by obtaining information away from the clinic.	
Please be honest and creative with your answers as every detail is important.	
For the following questions please focus on one medical condition that has affected you or people who are close to you.	
1. State the medical condition you want to focus on:	
2. What do you think is important to know to help prevent this condition?	
3. What do you think is important to know to help diagnose this condition?	
4. What do you think is important to know to help treat this condition?	
Please answer the following questions as if you were to wear a medical sensing device and list your preferences. Please do not think about the limitations of current technology and assume that anything is possible during the following five questions.	
5. What should the device look like?	
6. How should you control the device?	
7. Should the device be internal or external to the body?	
*Internal/External*	
**If internal;**	
Would you wear an external device in a potential life saving situation? *Yes/No*	
8. On which part of your body would you wear the device?	
9. How much would you spend on this wearable device annually?	

10. Do you currently use such a device?	*Yes/No*

**If Yes;**	
11. Could you describe or name the device?	
12. What is its main function?	
13. What do you like about the device?	
14. Is there anything that you don’t like about it?	

**If No;**	
15. Do you know any medical sensing devices that people wear?	*Yes/No*

**If Yes;**	
16. Could you describe or name the device?	
17. What is its main function?	
18. What do you like about the device?	
19. Is there anything that you don’t like about it?	

**Specific Questions**—After the open sections the reader should have their own thoughts on wearable devices and questions to identify specific desires relevant to today’s developers can be asked. Also this section verifies the findings from previous studies into the evaluation of current devices.

**Table:**

20. Would you wear a device that is visible to others?
*Yes/Maybe/No/Don’t know*
21. Would you wear a device that is concealed in your clothing?
*Yes/Maybe/No/Don’t know*
22. Would you wear an implanted device?
*Yes/Maybe/No/Don’t know*
23. Would you allow continuous monitoring 24 h a day?
*Yes/Maybe/No/Don’t know*
24. How long would you be willing to wear such a device over a 24 h interval?
*Less than 1 h/Less than 5 h/Less than 10 h/Less than 15 h/Less than 20 h/More than 20h*
25. How long should the device be able to work before the battery needs to be charged again?
*Less than 1 day /Less than 1 week/Less than 1 month/Less than 6 months /More than 6 months*
26. Would you allow data to be sent away and analysed or stored on a database?
*Yes/Maybe/No/Don’t know*
27. Would you consider wearing a device to monitor how active you are?
*Yes/Maybe/No/Don’t know*
28. Would you use a device that you had to apply yourself without assistance?
*Yes/Maybe/No/Don’t know*
29. Would you like the device to determine the level of your condition (e.g., mild or severe)?
*Yes/Maybe/No/Don’t know*
30. How long should it take to put the sensor device on?
*Less than 1 min/Less than 5 min/Less than 10 min/Less than 20 min/Less than 30 min/more than 30 min*
31. Would you wear a device that would tell the clinician if you did not wear it often enough?
*Yes/Maybe/No/Don’t know*
32. Would you spend time learning how to use a new wearable device?
*Yes/Maybe/No/Don’t know*
33. Would you wear an implant that has a sensor incorporated in it?
*Yes/Maybe/No/Don’t know*

**Table:**

34. On a scale of 1 to 10 how important are the following statements, with 10 being the highest.
A body worn device should...
...be comfortable
...be compact (light and small)
...be discrete
...be easy to attach to the body
...be recyclable
...be reliable
...be simple to operate (and maintain)
... blend in with different types of clothing to be worn with the device
...give instant feedback
...have clear and readable instructions
...increase the accuracy of current clinical procedures
...minimize incorrect use of the system
...motivate the people using it
...not affect normal daily behaviour
...not detach from patient unless needed
...provide clear and useful results
...provide positive feedback to patients
...reduce travel to clinics and hospitals
...speed up currently used clinical procedures

**Demographics**—This section will categorise the trends seen from the latter sections. Can leave blank if they want too.

**Table:**

35. Age:
36. Gender: *Male/Female*
37. Country of Birth:
38. County(UK)/City of Residence:
39. Marital Status: *Single/Married/Divorced/Widowed*
40. What is your highest educational level reached?
41. Employment / Occupation:
42. Annual Income: *below* *£25,000–$40,000–€30,000/above* *£25,000–$40,000–€30,000*
43. What medical condition(s) do you suffer from if any?
44. How affected are you by this condition?
*Very Affected/Slightly Affected/Not Much Affected/Not Affected*
45. How healthy would you rate yourself?
*Very healthy/Above average/Average/Below average/Very unhealthy*
